# Navigating challenges in prosthodontic rehabilitation after long‐term delay following fibula free flap reconstruction with a unique overlay removable prosthesis and large vertical dimension increase

**DOI:** 10.1111/jopr.70094

**Published:** 2026-01-21

**Authors:** David E. McReynolds, Advan Moorthy, Leo Stassen, Osama Omer

**Affiliations:** ^1^ Department of Restorative Dentistry and Periodontology, Division II Dublin Dental University Hospital, Trinity College Dublin Dublin Ireland; ^2^ Private Practice Dublin Ireland; ^3^ Royal College of Surgeons in Ireland Dublin Ireland

**Keywords:** fibro‐epithelial gingival hyperplasia, fibula free flap, implant overdenture, maxillofacial prosthodontics, occlusal plane disruption, sleeper implant, vertical dimension of occlusion

## Abstract

This case report highlights the experience of one maxillofacial surgical patient who delayed prosthodontic treatment for 7 years as they saved to self‐fund access to a subsidized prosthodontic service. Without the benefit of a restored opposing dentition for a prolonged period of time, maxillary occlusal plane disruption occurred as a downstream clinical consequence. To manage the complexities arising from delayed presentation, the prosthodontic team developed a highly tailored treatment plan to meet the patient's unique clinical needs, which included fabrication of customized healing abutments, an implant indexing device, and a definitive removable implant‐tooth‐supported occlusal overlay prosthesis, which incorporated an atypical increase in the vertical dimension of occlusion (VDO) of 12 mm. Following 68 months of follow‐up, the patient tolerated the increase in VDO well. Two minor technical complications occurred (denture tooth debond events) and one minor biological complication (localized peri‐implant mucositis) within the follow‐up period. This report underscores the role of timely prosthodontic involvement in interdisciplinary maxillofacial treatment pathways and advocates for the prioritization of equitable access to specialist prosthodontic services to mitigate preventable challenges in this vulnerable patient cohort.

The prosthodontist can serve as a key figure in the interdisciplinary planning and optimization of outcomes for patients undergoing maxillofacial reconstruction following resection of benign neoplasms, though anecdotally, barriers‐to‐access such a service remain commonplace.^1^ The purpose of this case report is to present a candid and transparent account of the challenges facing maxillofacial reconstructive patients and how the resourcefulness of a prosthodontic team might be called upon to overcome such obstacles and to advocate for and to champion the needs of this cohort of patients whose care could be optimized through access to a robust prosthodontic service.

## CASE REPORT

### Case history

In 2011, at the age of 21, a female patient presented to the maxillofacial unit of a national Irish hospital with a large, painless, and slow‐growing swelling located at the labial segment of the mandible (Figure 1a). While the patient was aware of the swelling for some time, she only presented for clinical assessment when her mandibular incisors became flared and mobile. Plain radiographic examination included a mandibular occlusal exposure and an orthopantomogram, which revealed a multilocular radiolucency that did not cause resorption of the adjacent teeth (Figure 1b,c). At the diagnostic work‐up phase, an ossifying fibroma (a locally aggressive but benign neoplasm) was identified following incisional biopsy. Given the extensive nature of this late presentation, a surgical treatment plan was embarked upon, which included partial mandibulectomy, single‐barrel vascularized osteocutaneous fibula free flap reconstruction, radial forearm skin graft, and dental implant placement. While ossifying fibroma is a benign neoplasm, the extent of surgical resection and microvascular reconstruction in this case paralleled those procedures more commonly associated with oncologic management of malignant lesions. Robust prosthodontic support was not sufficiently engaged, nor was an interim removable partial dental prosthesis successfully worn by the patient. A delayed referral to a prosthodontic service followed in 2018, 7 years after initial presentation, as the patient saved to afford prosthodontic care under a mixed public/subsidized‐private funding model.^2^, ^3^ At this juncture, clinically significant challenges faced the prosthodontic service and included maxillary occlusal plane disruption, poor anteroposterior distribution of the mandibular implants, and incomplete seating of the healing abutment at the 25 (lower right central incisor) implant site (Figure 1d).

**FIGURE 1:**
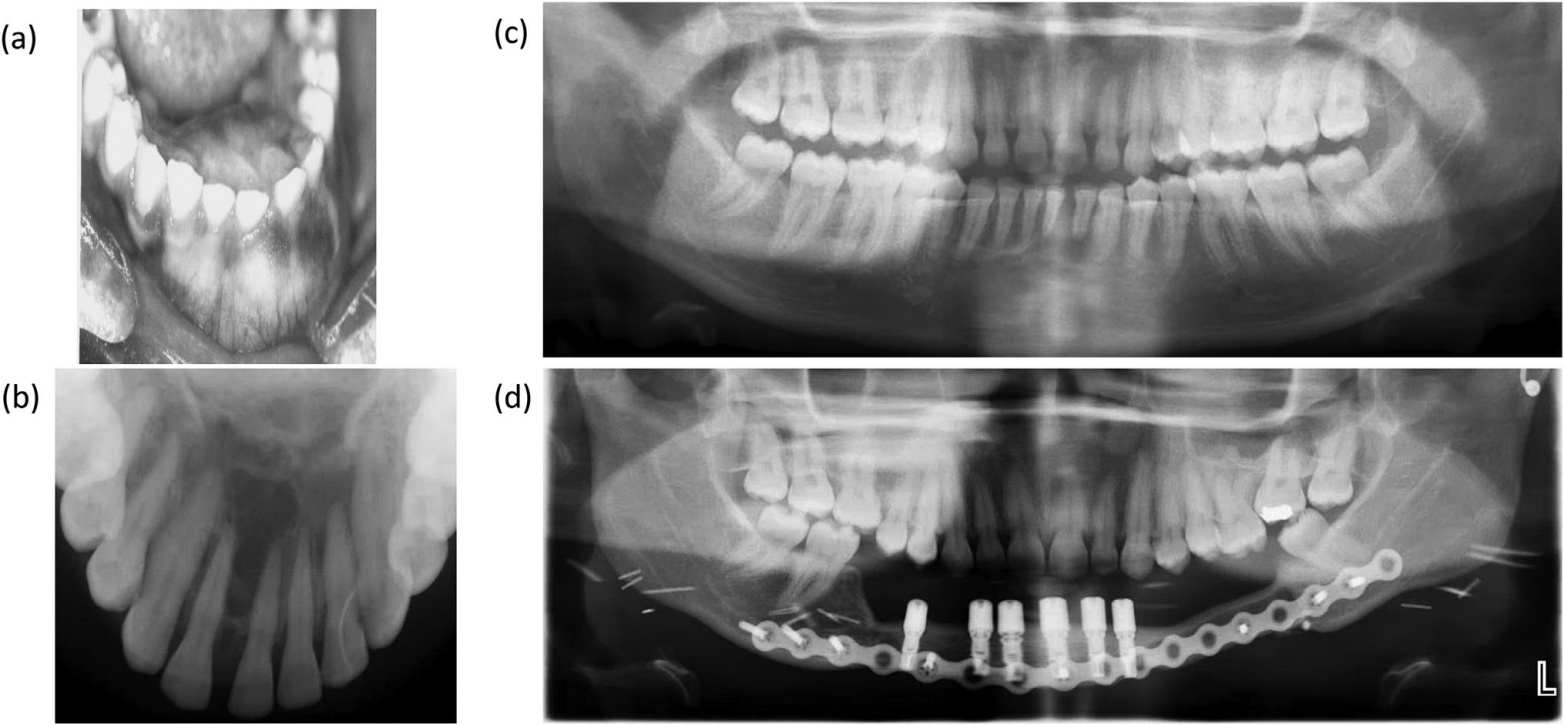
The patient, a 21‐year‐old female, presented at an oral and maxillofacial surgical unit in 2011 with an ossifying fibroma located at the anterior mandible (a‐c). Surgical management included partial mandibulectomy, fibula free flap reconstruction, forearm skin graft, and dental implant placement. A delayed referral for prosthodontic rehabilitation occurred 7 years later, in 2018, due to self‐funding delays (d).

### Prosthodontic assessment and record gathering

The initial prosthodontic assessment in 2018 involved intraoral and extraoral clinical photography (Figure 2a–e) and the fabrication of conventionally mounted study casts on a semi‐adjustable articulator (Figure 2f–h); the purpose of which was to identify the extent of the disruption of the static and dynamic factors of the patient's occlusal scheme and to help visualize proposed solutions to address these discrepancies.

**FIGURE 2:**
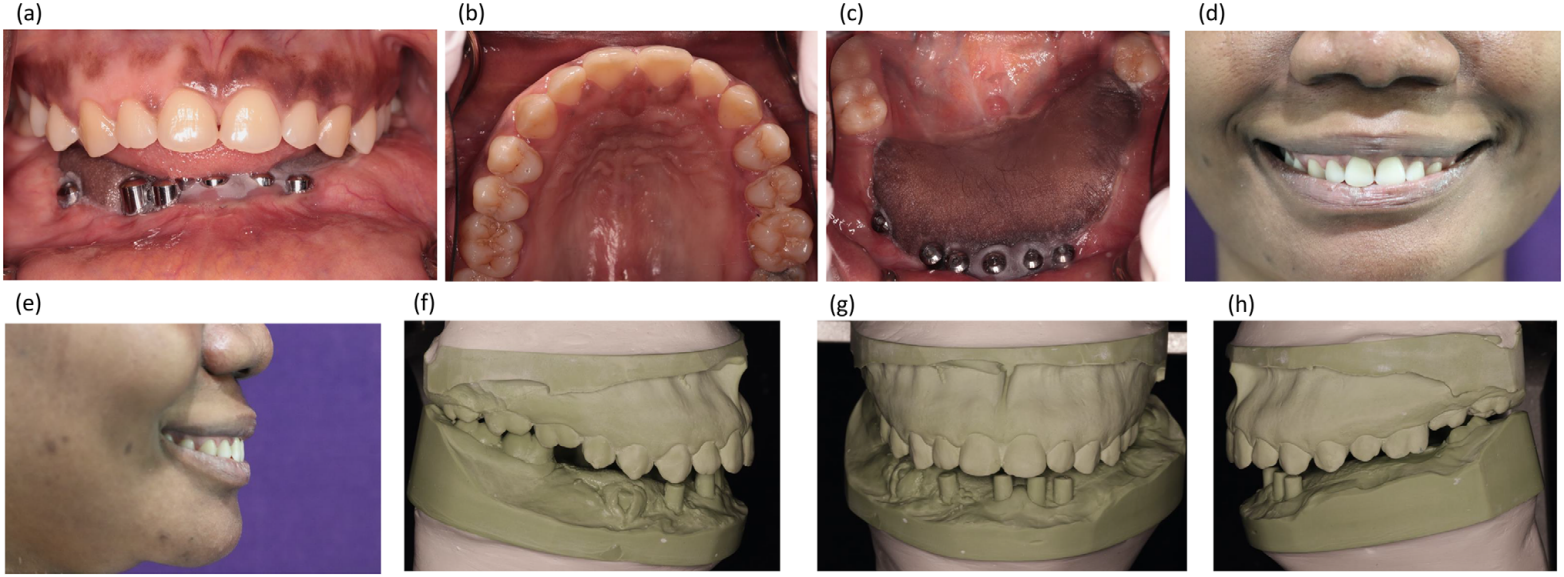
Following clinical prosthodontic assessment in 2018 (a–e) and fabrication of conventionally mounted casts (f‐h), the extent of over‐eruption of the maxillary dentition from tooth 4 to tooth 14, consequent loss of restorative space and vertical dimension of occlusion, and inversion of the lower lip were evident.

Upon prosthodontic assessment, the remaining maxillary and mandibular dentition was minimally restored, and caries risk was determined to be low. Periodontal pocketing was less than 3 mm in all sites, although oral hygiene was suboptimal with calculus present in all posterior segments. A clinically significant loss of restorative space and vertical dimension was evident spanning from tooth 4 (the maxillary right second premolar) to tooth 14 (the maxillary left first molar), invading the space formerly occupied by the opposing mandibular dentition. The radial forearm skin graft restored intraoral soft tissue contour at the lingual floor‐of‐mouth region, extending from tooth 18 (the lower left second molar) to tooth 30 (the lower right first molar) mediolaterally and to the buccal unattached oral mucosa anteroposteriorly, resulting in thin, displaceable, non‐keratinized, hair‐bearing cutaneous epithelium supporting dental implants at their lingual aspect and unattached non‐keratinized oral mucosa supporting dental implants at their buccal aspect. The hard tissue contours of the reconstructed neomandible were narrower superio‐inferiorly due to the single‐barrel approach to fibula free flap reconstruction. Six root‐form endosseous dental implants were placed from tooth 22 (the lower left canine) region to tooth 28 (the lower right first premolar) region, showing suboptimal anteroposterior spread, facial positioning at the lower right first premolar implant site, and slight divergent positioning of the implants. However, the fibula bone segment demonstrated stable osseointegration of the dental implants with no radiographic evidence of peri‐implant bone loss.

### Prosthodontic treatment plan

Following prosthodontic assessment and diagnostic record gathering, the prosthodontic treatment plan was separated into distinct phases with specific goals:

Interim prosthetic phase—The goal was to trial a removable complete overdenture supported by custom healing abutments and teeth on stabilized healthy soft tissues.

Definitive prosthetic phase—The goal was to translate the provisional removable design into an overdenture retained by a definitive implant‐supported bar and teeth that addressed the problems that were diagnosed at the initial assessment.

To meet the goals of prosthodontic rehabilitation in this instance, a removable implant‐tooth‐supported overdenture modality was selected, as such an approach would permit:

- Combined tooth‐implant support to compensate for poor implant anteroposterior distribution;
- An increased vertical dimension of occlusion (VDO) with a reorganized occlusal scheme to achieve full control over the posterior occlusion, thereby restoring occlusal stability and improving facial form;
- And importantly, being an implant‐supported removable dental prosthesis, this modality permits ease of thorough
  repeat examination of the oral cavity to provide the opportunity for early detection of neoplastic recurrence, if required, given the locally aggressive nature of the original pathology.

### Interim prosthesis phase

#### Acute treatment

One challenge arising in this case was sub‐optimal positioning and angulation of the implant at site 25, which precluded its deployment in the restoration. A cover screw was placed on this implant, and it was intentionally left in situ as a dormant implant as a strategic decision since restoration of this implant was neither feasible nor of net benefit in the overall prosthodontic treatment plan.^4^, ^5^

In situations involving mandibular vestibuloplasty, intraoral skin grafting, and implant placement, a known common complication is plaque‐induced gingival hyperplasia of fibroepithelial tissue adjacent to the transmucosal aspect of dental implants.^6^ In this case, implant placement was necessarily deep. However, the tallest‐available stock implant healing abutments were 7 mm in height (Figure 3a) and were not sufficiently tall to prevent such tissue reactions from engulfing access to the implant healing abutments. Therefore, custom healing abutments of 10 mm height were fabricated in milled polyetheretherketone (PEEK) (PEEK Dental Disc – 98 mm Round, Wieland, Ivoclar Vivadent, Schaan, Liechtenstein) (Figure 3b,c) and were fitted intraorally (Figure 3d) to permit relatively uncomplicated access to the implant heads and to stabilize the plaque‐induced tissue response.^7^ These custom healing abutments were subsequently used to support an interim removable complete overdenture.

**FIGURE 3:**
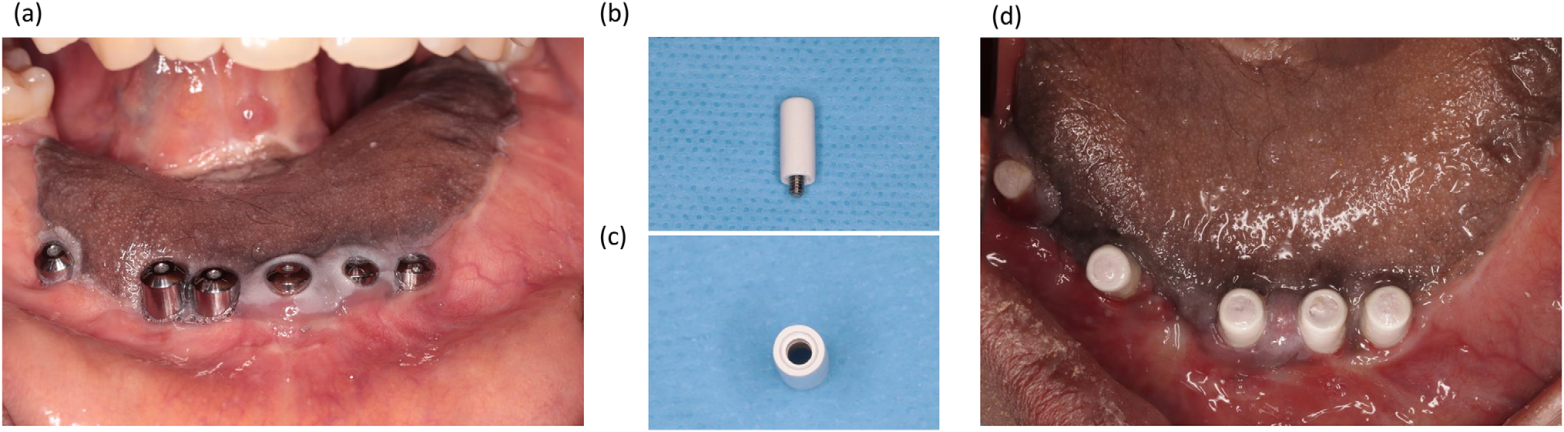
(a) Plaque‐induced fibro‐epithelial tissue swelling of the intraoral skin graft and non‐keratinized mucosa, which on occasion would engulf access to the implant healing abutments, despite the use of the maximum‐available 7 mm tall abutments (a. (b‐c) To overcome this problem, custom‐made polyetheretherketone non‐engaging abutments with 10 mm height were fabricated and fitted intraorally (d) with a view towards stabilising such tissue responses.

#### Fabrication of a verified implant master cast

Another challenge arising from the necessary depth of implant placement manifested in the unstable transfer of implant impression copings from their intraoral arrangement to a gypsum cast. To overcome this difficulty, a primary implant cast (known to be of sub‐optimal accuracy) was produced with a view toward fabricating a custom milled cobalt‐chrome implant indexing device intended to act as both a set of custom tall implant impression copings and a verification jig (Figure 4a,b). The custom cobalt‐chrome implant indexing device was sectioned and placed intraorally, with complete seating verified using orthopantomogram radiography (Figure 4c) (as the grafted floor‐of‐mouth anatomy prohibited placement of intraoral films to sufficient depth); the device was united intraorally with autopolymerizing acrylic resin (GC Pattern Resin, GC Europe, Haasrode‐Leuven, Belgium) (Figure 4d) and picked up in polyether (3M Impregum 3M Ireland, Dublin, Ireland) into a custom impression tray using a closed‐mouth technique (Figure 4e). This process permitted fabrication of an accurate, reliable, and verified secondary implant master cast, from which definitive framework fabrication could occur.^8^, ^9^, ^10^, ^11^

**FIGURE 4:**
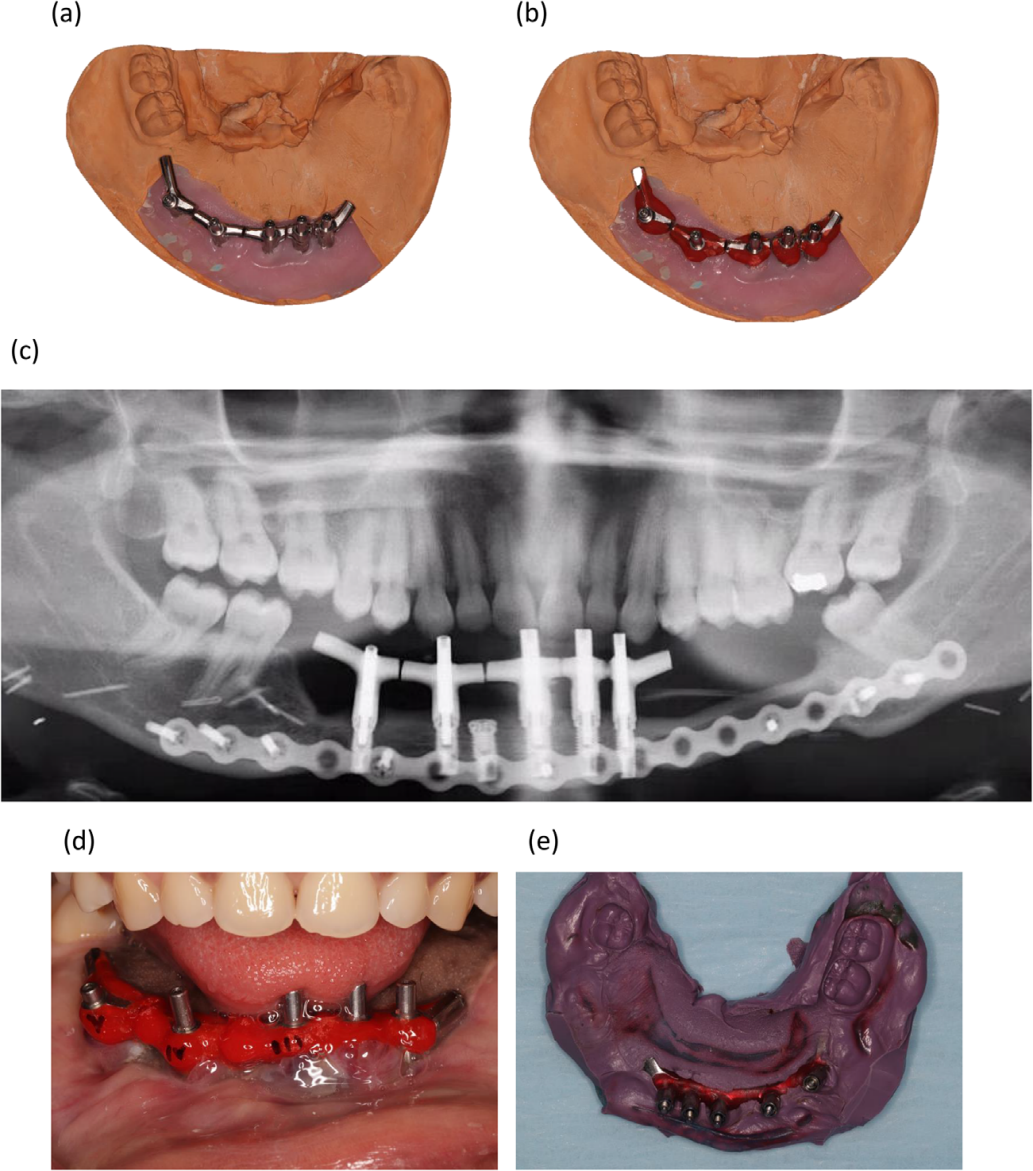
Conventional implant impression copings could not be relied upon for stable transfer from the mouth, given the necessary depth of implant placement. A custom‐fabricated milled cobalt‐chrome implant indexing device based on a primary implant cast was developed, sectioned, and splinted intraorally with autopolymerizing acrylic resin and picked‐up in a conventional polyether impression, following verification of its complete seating with a panoramic radiograph.

A stabilized wax occlusion rim was contoured according to classic prosthodontic principles to establish the vertical dimension necessary to generate sufficient restorative space and to re‐establish occlusal control in this case, without unduly compromising closest speaking space, sibilant enunciation, facial balance, aesthetics, and lip competence (Figure 5a–d).^12^, ^13^ This resulted in the generation of approximately 4 mm of restorative space at the level of the second molar, which was required for cobalt‐chrome overlays, heat‐processed denture acrylic (ProBase Hot [Ivoclar Vivadent, Liechtenstein]), and highly cross‐linked denture teeth (SR Phonares II [Ivoclar Vivadent, Liechtenstein]) with uncompromised anatomical form (Figure 5e,f). To ensure stability of the wax occlusion rim, which in turn would produce a comfortable, reproducible, and recordable inter‐occlusal record, the light‐polymerized record base (Triad VLC, Dentsply Sirona, North Carolina, USA) was designed to derive support and stability from the custom, tall PEEK healing abutments, as described.^14^

**FIGURE 5:**
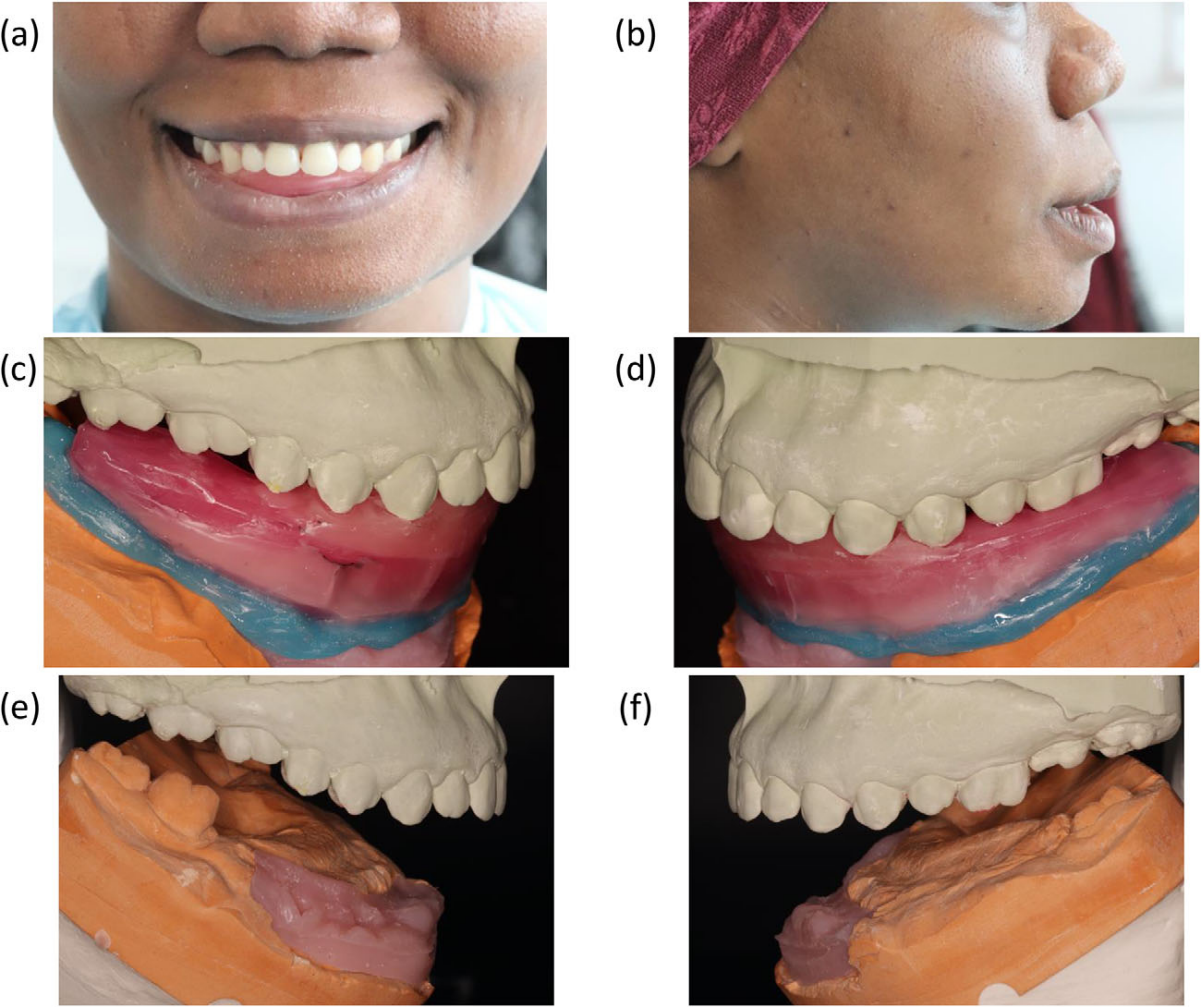
(a‐d) Restoration of the patient's vertical dimension of occlusion and generation of restorative space was developed with a conventional contoured occlusion rim on a light polymerized stabilized record base. (e‐f) 4 mm of restorative space at the second molar region was deemed clinically necessary to fabricate a stable and predictable restoration.

#### Trialing the increased VDO

Given the extent of the planned rise in VDO in this case, the patient was asked to wear a polymethylmethacrylate (PMMA) (Telio CAD LT 98.5–25 mm, Ivoclar Vivadent, Schaan, Liechtenstein) trial removable complete dental prosthesis for 3 months, which was created on the basis of the contours of the patient‐ and clinician‐approved wax trial set‐up (Figure 6a,b). After the trial period, the patient reported no issues and appeared to tolerate the increased VDO very well (Figure 6c).

**FIGURE 6:**
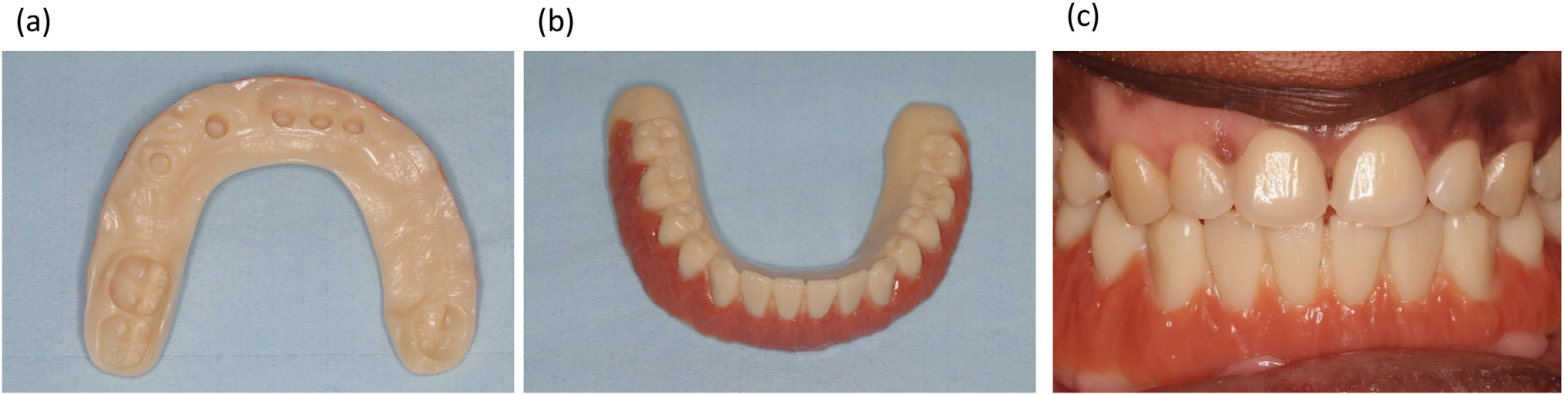
Although the increase in vertical dimension was guided by classic prosthodontic principles, good practice dictates that such a large increase in VDO is trialled to ensure the patient can tolerate such an adjustment. In this instance, a copy‐milled PMMA removable complete dental prosthesis based on the contours of a patient‐approved wax trial prosthesis was fabricated (a‐b). The patient successfully wore the prosthesis for 3 months before proceeding to the definitive restorative phase. (c).

#### Definitive prosthetic phase

The team proceeded with the final fabrication steps for the definitive implant‐tooth‐supported complete overdenture. Specifically, the definitive restoration in this case was a milled titanium meso‐structure LOCATOR® (Zest Dental Solutions, California, USA) bar, a corresponding double‐structure telescopic sleeve (Panthera Double Structure, Quebec, Canada), and a cobalt‐chrome framed supra‐structure with laser‐welded overlays to derive support from the natural dentition, establishing bilateral simultaneous even contacts in the reorganized maximum intercuspation position and canine guidance on laterotrusive excursive movements of the reconstructed neomandible (Figure 7a–f).^15^

**FIGURE 7:**
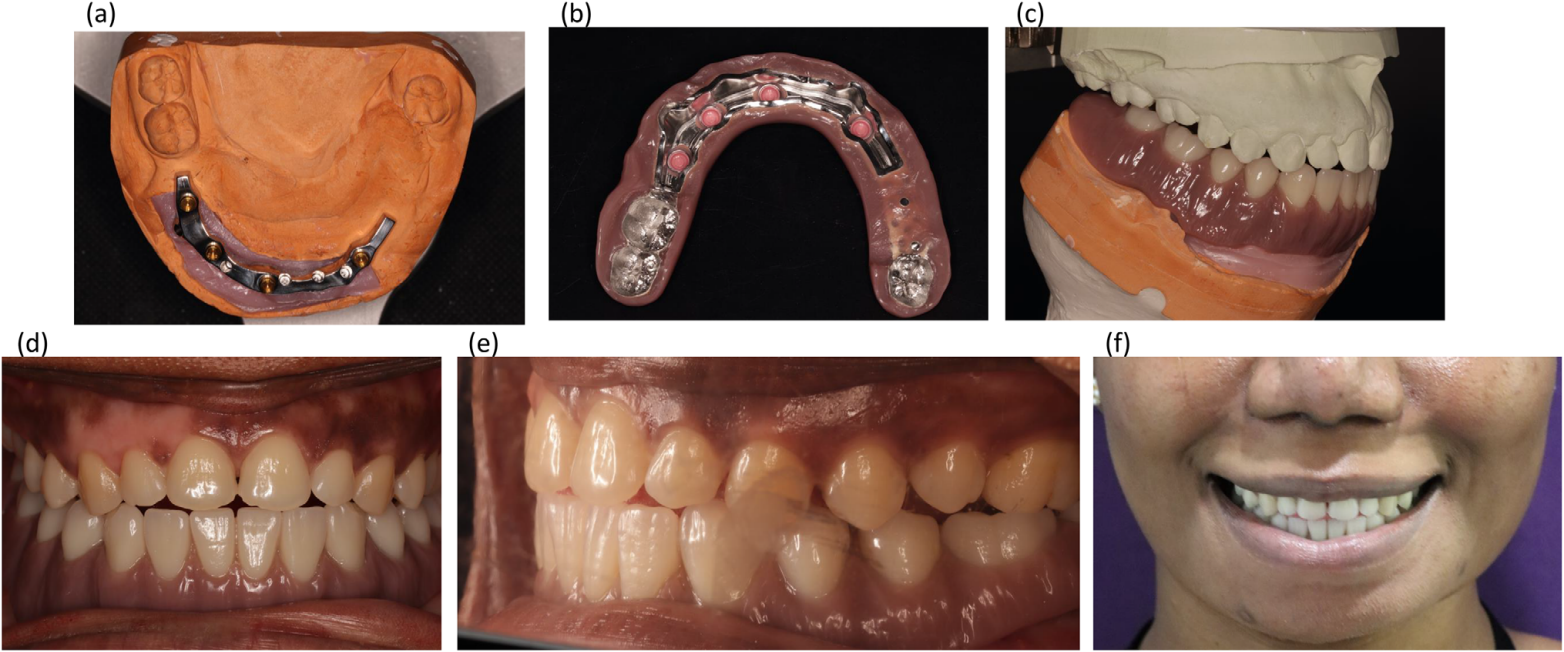
Definitive restoration with a milled LOCATOR® bar and telescopic sleeve double‐structured removable complete implant‐tooth supported overdenture. Given the idiosyncrasies of this case, tooth‐supported overlays on the remaining mandibular molars were applied to successfully establish an acceptable occlusal outcome and to leverage combined tooth‐implant support.

## PROSTHODONTIC MAINTENANCE AND FOLLOW‐UP

On completion of the definitive prosthetic phase, the patient was advised that an intraoral removable prosthesis of this nature should be removed from the mouth at night, every night, and the prosthesis should be mechanically cleaned with a soft‐bristled brush and low‐abrasive denture dentifrice.^16^ The patient was advised that care should be taken with immersion disinfection, particularly with the avoidance of sodium hypochlorite as a denture disinfectant, as corrosion of metal parts and degradation of retentive nylon elements are likely to occur upon such an exposure.^16^, ^17^ An implant‐tooth‐supported mandibular occlusal device was fabricated and fit for nighttime use, to protect the implant mesostructure from overloading conditions and to maintain occlusal stability (Figure 8a,b).^15^ The patient was provided with a LOCATOR® Core Tool (Zest Dental Solutions, California, USA) and Clear High‐Retention Standard Range Nylon Replacement Males (Zest Dental Solutions, California, USA) with education on how to replace retentive nylon inserts without the need for professional assistance. The patient was advised that replacement of nylon retentive elements should be expected to be necessary at 12–24 month intervals. Subsequently, the patient was recalled for regular review with a maxillofacial prosthodontist once yearly, and with a dental hygienist for supportive periodontal and peri‐implant therapy three times per year, where review included a focus on continued implant, mesostructure, and prosthesis‐level professional and at‐home cleansability, as well as the prevention of peri‐implant disease.^18^

**FIGURE 8:**
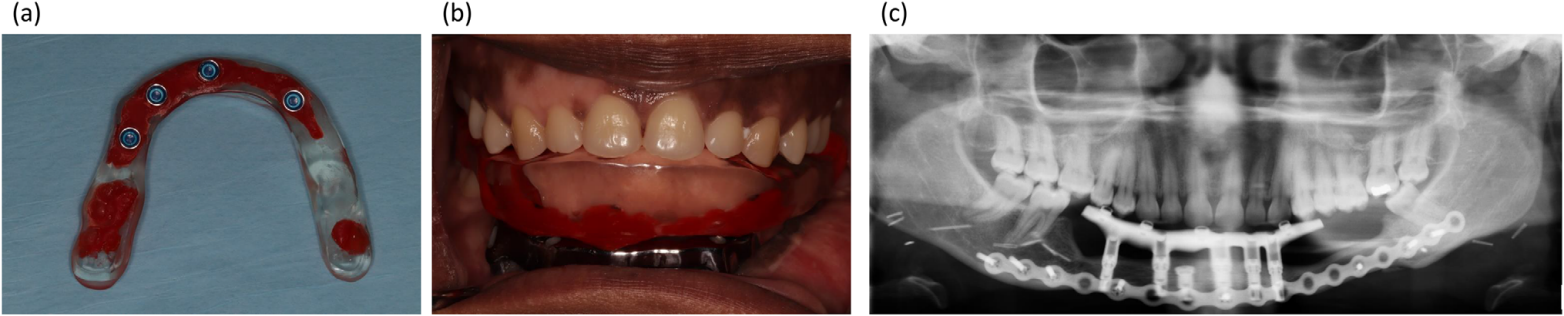
(a‐b) An implant‐tooth supported mandibular occlusal device was fabricated to protect the implant mesostructure from overloading conditions and to maintain occlusal stability during night time). (c) Post‐operative panoramic radiography at 65 month review confirms stability of the treatment outcome.

Upon long‐term review, it was found that the patient elected to replace their retentive nylon inserts approximately once every 6 months with the desire of achieving sustained high levels of retention security, and the patient could do so independently. Two episodes of technical complications occurred involving debonding of denture teeth at the 23 and 26 sites: once at 24 months post‐fit and again at 68 months post‐fit at the same location. Each episode of denture tooth debond was managed with a same‐day laboratory denture repair using autopolymerizing PMMA (ProBase Cold [Ivoclar Vivadent, Liechtenstein]). One biological complication episode involving peri‐implant mucositis and suppuration, affecting implants at the 23 and 25 sites occurred at 49 months post‐fit. This complication was managed with localized professional debridement and 0.2% chlorhexidine (PerioKin Mouth Rinse, Laboratorios Kin, Barcelona, Spain) irrigation, along with tailored oral hygiene advice for maintenance of at‐home plaque control, following which complete resolution of the peri‐implant mucositis occurred. An orthopantomogram was made at 65 months post‐fit of the definitive prosthetic (Figure 8c), which displayed overall stability of the treatment outcome, with no evidence of dental caries, periodontitis, peri‐implantitis, or graft pathology. The patient showed adaptation to the increased vertical dimension of occlusion without complication on all review appointments.

## DISCUSSION

Without the benefit of sufficiently robust prosthodontic planning and continuity of access to a prosthodontic service in the immediate years post‐surgery, this case represents a cautionary tale regarding the clinically significant challenges that may arise when navigating definitive and comprehensive prosthodontic rehabilitation in the management of maxillofacial surgical cases. It is notable in this case that an unplanned and uncontrolled disruption of the maxillary occlusal plane, which occurred over the course of 7 years during self‐funding delays (despite access to a reduced‐cost prosthodontic service), ultimately mandated a complex reorganized approach to occlusal rehabilitation, which otherwise would not have been necessary if timely restoration of an opposing dentition had occurred. In this case, it is thought that eruption of the maxillary dentition occurred in response to the maxillary dentition remaining unopposed over a prolonged period of time. Such cost sensitivities are understood to be a global phenomenon that represent a barrier‐to‐care for many maxillofacial reconstructive patients.^1^, ^19^, ^20^

In this case, the clinicians could have restored the dental implants with intermediate multi‐unit abutments in order to reposition the restorative platform closer to tissue level, rather than at the level of the implant heads. This approach would have simplified the need for repeated soft tissue manipulation and could have simplified implant mastercast fabrication. Additionally, previous research has indicated that multi‐unit abutments may act as a “mechanical fuse,” in full‐arch implant cases, which may shift prosthetic complications to the prosthetic screw and away from the implant head.^21^ Despite these potential advantages, a clinical decision was made to streamline the restorative design at the implant‐restorative interface to reduce complexity, interfaces, and the number of prosthetic components in the definitive restoration.

A systematic review of the literature has supported the safety and predictability of increasing VDO by up to 5 mm.^22^ However, in this case, an increase of 4 mm at the second molar region was deemed clinically necessary to fabricate a stable and predictable restoration (Figure 2f–h and Figure 5e,f), which represents an increase of VDO of approximately 12 mm at the level of the incisal pin of the articulator. Increasing the VDO excessively risks initiation or exacerbation of temporomandibular joint dysfunction, facial pain, tenderness of the muscles of mastication, hyperactivity of the masticatory muscles, and an increase in bite force.^23^ Patient‐reported symptoms of psychological stress, headache, awareness of clenching and grinding, muscle and joint fatigue, soreness of teeth, cheek biting, and difficulties with chewing and speech may arise.^24^, ^25^ It is noteworthy that in this case, no such sequela occurred, and the patient tolerated this large, atypical increase in VDO without incident, with long‐term follow‐up at 68 months.

## CONCLUSION

Prosthodontic ingenuity was called upon at various stages to address the unique challenges facing this patient. This case highlights how resource constraints necessitated pushing the boundaries of prosthodontic techniques to arrive at a satisfactory outcome of maxillofacial prosthodontic reconstruction. Specifically, a deviation from standard protocol was implemented by increasing VDO to address case‐specific requirements; a necessary response to the complex circumstances, which were largely thought to be shaped by a barrier‐to‐access faced by this patient. This case report reaffirms the assertion that engaging a prosthodontic service at an early stage can minimize the need for treatment throughout the life course of the maxillofacial reconstructive patient.^26^

## CONFLICT OF INTEREST STATEMENT

The authors declare no conflicts of interest.
